# Sleep, Autonomic Nervous Function and Atherosclerosis

**DOI:** 10.3390/ijms20040794

**Published:** 2019-02-13

**Authors:** Manabu Kadoya, Hidenori Koyama

**Affiliations:** Department of Internal Medicine, Division of Diabetes, Endocrinology and Metabolism, Hyogo College of Medicine, Nishinomiya, Hyogo 663-8501, Japan; mkadoya@hyo-med.ac.jp

**Keywords:** sleep, autonomic nervous dysfunction, atherosclerosis, obesity, fatigue, cardiovascular disease

## Abstract

Behavioral and psychosocial factors related to development of cardiovascular disease have been gaining increased attention. Notably, sleep is considered to be one of the most important behavioral factors involved in progression of atherosclerosis and cardiovascular events, with autonomic nervous function a potential mechanism. Several studies have shown associations of sleep and autonomic dysfunction with major surrogate markers of atherosclerosis, such as carotid intima-media thickness and arterial stiffness. Endocrinological, immunological, oxidative, inflammatory, and metabolic responses, as well as endothelial dysfunction may mediate the effects of the autonomic nervous system. For this review, we examined recent findings related to sleep, autonomic nervous dysfunction, and atherosclerosis, with the aim of understanding the involved pathophysiological mechanisms.

## 1. Introduction

**Figure 1:**
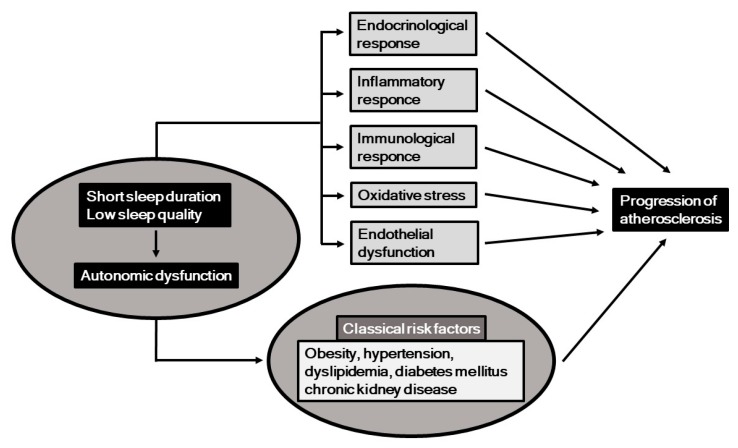
Short sleep duration and low sleep quality, along with resultant autonomic nervous dysfunction may induce progression of atherosclerosis, potentially through endocrinological, immunological, inflammatory, and oxidative response, and endothelial dysfunction.

Classical cardiovascular risk factors, including obesity, hypertension, dyslipidemia, diabetes mellitus, and chronic kidney disease (CKD), are established predictors of atherosclerosis and cardiovascular disease (CVD) [1,2,3]. Recently, behavioral and psychosocial factors have been gaining increased attention in regard to development, prevention, and treatment of CVD [4], with short sleep duration and low sleep quality shown to be important behavioral factors that may be involved in its occurrence [5]. In this context, potential mechanisms associated with short sleep duration and low sleep quality include autonomic nervous function, and a previous study showed a strong association between those in a general population [6]. Additionally, autonomic nervous dysfunction has also been found to be a risk factor for CVD [7]. Potential mechanisms related to the associations among sleep, autonomic nervous function, and progression of atherosclerosis are summarized in Figure 1. It has been shown that short sleep duration, low sleep quality, and autonomic nervous dysfunction are associated with several risk factors for atherogenesis, including endocrinological, immunological, oxidative, inflammatory, and metabolic responses, as well as endothelial dysfunction.

Sleep duration and quality can be measured by subjective methods, including self-reported questionnaires, the Epworth Sleepiness Scale (ESS), and Pittsburg Sleep Quality Index (PSQI), as well as by objective methods, including actigraphy [8,9], while apnea-hypopnea during sleep can be quantitatively determined using polysomnography or apnomonitor results [10,11]. Standardized autonomic nervous function tests including Valsalva maneuver, hyperventilation, standup tilt test, cold pressor, isometric handgrip, and heart rate variability (HRV) are also performed [12,13], among which HRV is a practical method to assess impaired autonomic nervous function in clinical settings [7].

Several studies have found relationships of short sleep duration, low sleep quality, and autonomic dysfunction with carotid intima-media thickness (IMT) and brachial-ankle pulse wave velocity (baPWV), which are major surrogate markers of atherosclerosis and established predictors of cardiovascular events [14,15]. We recently reported associations among sleep quality, autonomic nervous function, carotid IMT, baPWV, and nocturnal hypertension in subjects with risk factors for atherosclerosis who participated in the Hyogo Sleep Cardio-Autonomic Atherosclerosis (HSCAA) study [11,16,17,18]. The aim of this review was to summarize findings regarding the impact of sleep and autonomic nervous function on atherosclerosis, and elucidate their underlying mechanisms related to the progression of atherosclerosis.

## 2. Sleep and Atherosclerosis

Several epidemiological studies have suggested associations of short sleep duration, low sleep quality, and obstructive sleep apnea (OSA) with atherosclerosis. Table 1 summarizes reports of patients with atherosclerotic risk factors regarding the association between sleep and surrogate markers of atherosclerosis, carotid IMT, and baPWV, though the majority of the cohorts examined were relatively small in size. As for subclinical atherosclerosis in 86 elderly patients (73.6 ± 4.9 years old), carotid IMT in subjects with shorter sleep duration (<5 h) examined with the PSQI was found to be higher than those with the reference number of sleep h (>7 h) [19]. In a study of 201 elderly patients (79.9 ± 6.4 years old), carotid IMT was also shown to be higher in those with subjective persistent insomnia than in those with no insomnia [20]. Yoda et al. assessed using objective sleep quality and elector-encephalography findings, and reported a significant correlation between carotid IMT and rapid eye movement latency in 63 patients with type 2 diabetes mellitus [21]. Furthermore, in the HSCAA study with a relatively large number of patients (*n* = 330) with cardiovascular risk factors, we showed that apnea-hypopnea index (AHI) assessed by an apnomonitor and poor sleep quality determined with actigraphy were each significantly and positively associated with carotid IMT and plaque score [11]. Regarding arterial stiffness, subjective low sleep quality was reported to be correlated with higher baPWV in 724 patients with type 2 diabetes [22], while our recent study demonstrated that low sleep quality is significantly associated with impaired nocturnal blood pressure fluctuations, a risk factor for arterial stiffness [17]. Arterial stiffness was also shown to be independently associated with obstructive sleep apnea in 127 patients with ischemic stroke [10].

Until recently, no prospective study examined the association of sleep duration or quality with atherosclerotic progression in patients with atherosclerotic factors. Our report presented in 2018 of a 3-year longitudinal investigation (*n* = 306) in association with the HSCAA study was the first to show a relationship of low sleep quality with progression of baPWV [18]. Those findings indicated that poor sleep quality is associated with progression of arterial stiffness independent of other cardiovascular risk factors, including ambulatory blood pressure, apnea-hypopnea, and cardiac autonomic function, in patients with cardiovascular risk factors.

Some largescale studies examined associations of subjective and objective sleep duration, and quality with carotid IMT and baPWV in healthy general populations (Table 2). In an investigation of 617 middle-aged healthy subjects (37–52 years old), Sands et al. showed that objective shorter sleep duration was associated with greater carotid IMT [23]. However, it is important to note that the association of subjective sleep duration with carotid IMT was U-shaped in that healthy population. Wolff et al. reported that both longer (>11 h) and shorter (<5 h) sleep duration was associated with increased risk of atherosclerosis as compared to the reference sleep duration (7–8 h) in a general population (*n* = 2383) [24]. Abe et al. also queried 2214 general population subjects and showed that a longer sleep duration (>7 h) was significantly correlated with the incidence of carotid artery atherosclerosis as compared with a duration of 6 h [25]. Additionally, several studies revealed that longer sleep duration has an association with the incidence of stroke and cardiovascular mortality [26,27], while several largescale studies found associations of sleep duration and quality with baPWV. Importantly, the association between sleep duration and baPWV also had a U-shape in a manner similar to the association of sleep duration with carotid IMT. In a large general population study (*n* = 18,106), Kim et al. reported that both longer (>8 h) and shorter (<5 h) sleep durations were associated with higher baPWV as compared with recommended sleep time (7 h) [28]. Yoshioka et al. also showed that daily sleep duration (>9 h) was associated with elevated baPWV in 4268 employees [29]. Also, in 3508 males in the general population, Tsai et al. found an association between long sleep duration and increased baPWV [30]. More recently, low sleep quality was shown to be associated with subclinical coronary atherosclerosis, as assessed by cardiac computed tomography [31].

## 3. Potential Mechanisms underlying Association of Sleep with Atherosclerosis Progression

**Figure 2:**
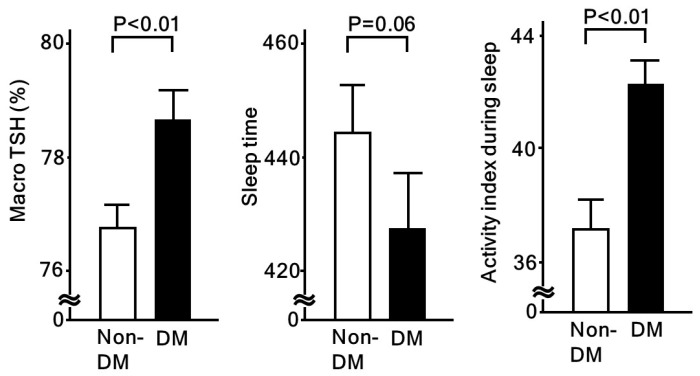
Macro-TSH and sleep quality assessed by actinography in patients with and without diabetes. Macro-TSH is significantly associated with low sleep quality [34]. When compared between non-diabetic (non-DM) and diabetic (DM) patients, all macro-TSH and activity index during sleep (high values represent poor sleep quality) were significantly higher in DM patients. Values shown in each column represent the mean ± standard error. Student’s *t*-test was used for analyses.

Short sleep duration and low sleep quality can have strong effects on several aspects of endocrinological, immunological, and metabolic responses. Both are known to disturb the daily rhythm of the hypothalamic-pituitary-adrenal axis. Following activation of that axis, resultant higher cortisol levels are associated with insulin resistance, cardiovascular risk factors, and coronary heart disease [32]. Also, Lee et al. showed that testosterone, a cortisol-linked stress hormone, is associated with coronary artery calcium (CAC) and carotid IMT [33]. In our study, we reported a relationship of low sleep quality with higher serum macro TSH (Table 3), which was found to be regulated in a manner distinct from free TSH, potentially due to an altered glycosylation structure [34]. In our subjects, serum macro TSH levels and sleep physical activity index values (higher value indicating poor sleep quality) were higher, while total sleep time was lower in patients with diabetes, as compared to those without (Figure 2). On the other hand, no such significant differences were found when the patients were categorized by the presence or absence of hypertension or dyslipidemia. Serum macro TSH levels were also shown to be significantly associated with fasting glucose, HbA1c, and homeostasis model assessment for insulin resistance (HOMA-R). Together, these results suggest that sleep quality is deeply related to altered endocrinological responses, which may be involved in progression of atherosclerosis through modulation of metabolic status.

It has also been reported that oxidative stress, subclinical systemic inflammation, and endothelial dysfunction may mediate the effects of short sleep duration and low sleep quality [35,36,37]. Short sleep duration and low sleep quality have been shown to induce a proinflammatory state, characterized by increased levels of several cytokines, including IL1β, TNFα, IL6, and IL17 [38,39,40]. Another study of rodents noted that chronic sleep fragmentation may induce morphological vessel changes, which are characterized by disruption and disorganization of elastic fibers, and increased recruitment of inflammatory cells [41]. An epidemiologic study found that short sleep duration (4 h) contributes to endothelial dysfunction, as measured by flow-mediated brachial artery vasodilatation (FMD) [42]. Other reports have shown that oxidative stress, such as total antioxidant capacity (TAC) and 8-hydroxy-2-deoxyguanosine (8-OHdG), is elevated in patients with OSA. Oxidative stress may be involved in dysregulation of collagen and elastin fibers of the vascular wall, leading to increased arterial stiffness [36,43]. Furthermore, study findings have indicated that OSA can lead to endothelial dysfunction and arterial disease [37], in which intermittent hypoxia and intrathoracic pressure changes may be involved.

In addition to progression of atherosclerosis, several have reported close associations of sleep condition with obesity, including epidemiologic examinations that found an association between short sleep duration and weight gain [44,45,46]. Potential mechanisms underlying this relationship are feeding behavioral changes and dysregulation of the neuroendocrine system, including the leptin-ghrelin system. Fang et al. conducted a human study that showed that sleep deprivation leads to increased fat intake through brain connectivity from the dorsal anterior cingulate cortex to the putamen and anterior insula [47]. Taheri et al. also presented results showing that short sleep duration may reduce leptin and induce ghrelin [48]. Moreover, in basic research findings, chronic sleep fragmentation was indicated to induce hypothalamic endoplasmic reticulum stress, which is associated with leptin resistance, alters eating behavior, and leads to weight gain [49]. A recent study found that acute sleep loss is attributable to epigenetic changes in adipose tissue and skeletal muscle, which are the result of an alteration of metabolic fuel utilization [50]. In that study of individuals with sleep loss, down-regulated proteins in skeletal muscles were shown to include genes involved in glycolysis, such as phosphoglycerate kinase 1 (PGK1), whereas the protein is up-regulated in adipose tissue. It was also demonstrated that alterations in circadian rhythm may be involved, since protein levels of the core clock component BMAL1 were significantly higher in skeletal muscle. Indeed, significant roles of circadian rhythm and BMAL1 in metabolic utilization have been shown in animal studies [51,52].

**Figure 3:**
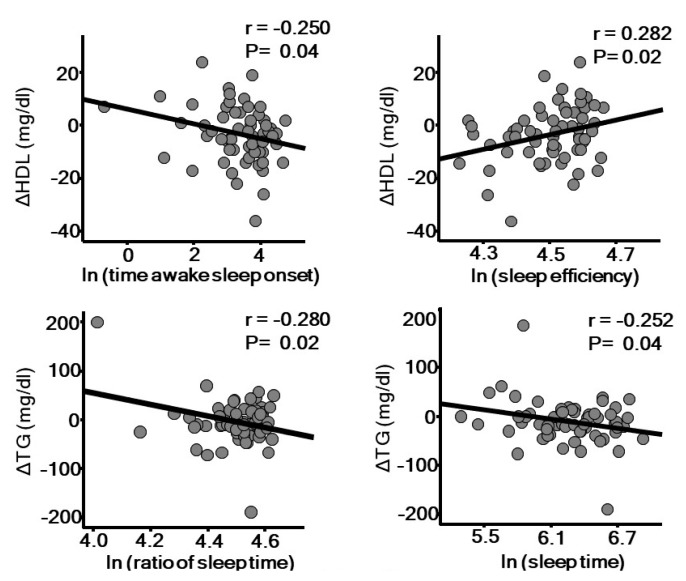
Sleep quality assessed by actigraphy is significantly associated with annual changes of high density lipoprotein (HDL) cholesterol and triglyceride level. The parameters of sleep quality were natural logarithm-transformed (ln) to achieve a normal distribution. *r*: Pearson’s correlation coefficient.

A longitudinal study reported that low sleep quality due to insomnia increases the risk for hypertension [53]. The association between OSA and hypertension has been well established by findings of several pathophysiological and epidemiological studies, in which autonomic dysfunction, including sympathetic and parasympathetic imbalance, appears to be involved. Furthermore, cross-sectional studies have also shown a significant association between OSA and dyslipidemia [54,55]. A recent review by Barros [56] elegantly summarizes potential mechanisms related to sleep and dyslipidemia. Intermittent hypoxia due to OSA up-regulates hypoxia-induced factor-1 (HIF-1), which might be involved in lipolysis in adipose tissue lipolysis, lipid synthesis in the liver favoring secretion of very low-density lipoprotein (VLDL), and delayed clearance of triglyceride-rich lipoprotein. Excessively produced reactive oxygen species (ROS) may be involved in generation of an oxidized form of LDL cholesterol, which is known to have a more atherogenic form. Indeed, higher levels of oxidized LDL cholesterol have been found in patients with OSA [57]. Dysfunction of high-density lipoprotein (HDL) has also been suggested in OSA patients [58], which may be mediated through increased noradrenaline and cortisol secretion [59,60]. In our 1-year prospective study, we also found that low sleep quality is associated with worsening of dyslipidemia (Figure 3). Objectively measured poor sleep quality and short sleep duration are each associated with a decrease in HDL cholesterol and increase in triglyceride level, independent of sleep apnea-hypopnea index. Short sleep duration has also been shown to be associated with increased prevalence of diabetes and impaired glucose tolerance [61,62]. A 15-year longitudinal prospective study showed that difficulty with falling asleep or regular use of hypnotics is associated with diabetes incidence in middle-aged men [46]. Moreover, several epidemiological studies have found that OSA is an independent risk factor for development of diabetes [63], in which autonomic dysfunction, oxidative stress induced by intermittent hypoxia, and inflammation may be involved. The associations between OSA and atherosclerotic risk factors may not be universal among ethnic groups. Recent papers based on Multi-Ethnic Study of Atherosclerosis (MESA) results showed that low sleep quality is associated with hypertension or peripheral artery disease (PAD) in blacks, but not in whites or Hispanics. Potential mechanisms of this heterogeneity include differences in salt sensitivity and diet quality among ethnicities [64].

## 4. Autonomic Nervous Function and Atherosclerosis

HRV provides a practical means to assess impaired autonomic nervous function in clinical settings by use of continuous electrocardiographic records, as those reflect sympatho-vagal balance and parasympathetic nervous activity.

It has been shown that reduced HRV predicts all-cause mortality and cardiovascular events [7], while that has also been recognized in patients with myocardial infarction [65], diabetes mellitus [66], and short sleep duration or low sleep quality [6,11]. Table 4 summarizes the limited numbers of studies presented thus far that have examined the relationships of HRV with carotid IMT and baPWV in specific patient groups [11,67,68,69,70,71,72]. In a longitudinal study, Gottsater et al. showed an association of low HRV with progression of carotid IMT in 61 type 2 diabetic patients over a period of 3–4 years [67]. Furthermore, Melillo et al. reported that low HRV was significantly associated with carotid IMT in 200 hypertensive patients [68]. In the HSCAA study, we found that HRV was associated with carotid IMT in patients with cardiovascular risks, independent of sleep quality and apnea-hypopnea [11]. Pizzi et al. showed an inverse association of carotid IMT or inflammatory markers (CRP, IL-6) with HRV in 391 depressed subjects with coronary risk factors [69], while Ulleryd et al. reported mutual relationships among HRV, inflammatory markers (CRP, white blood cell count), and carotid atherosclerosis in 124 men over 40 years old [70]. Together, these results suggest that inflammation plays an important role in the association of autonomic dysfunction with atherosclerosis. Recently, Pereira et al. reported that HRV noted during a deep breathing test was significantly and negatively correlated with carotid IMT in 101 patients with atherosclerotic risk factors [71]. As for arterial stiffness, Jaiswal et al. found that HRV was significantly and negatively associated with baPWV in 344 patients with type 1 diabetes [72], though no prospective studies that investigated this association have been presented.

## 5. Potential Mechanisms underlying Association of Autonomic Nervous Dysfunction and Progression of Atherosclerosis

Autonomic nervous dysfunction can cause alterations in immunological response and endothelial dysfunction, which eventually could lead to progression of atherosclerosis, while an increased level of inflammatory cytokines may mediate the effects of autonomic nervous dysfunction on atherosclerosis progression [69,70]. Similar to the association between low sleep quality and atherosclerosis, augmented recruitment of inflammatory cells into vessel walls induces morphological changes in vessel cells. Another recent study suggested that autonomic dysfunction dysregulates neurotransmitters, such as norepinephrine (NE), adenosine triphosphate (ATP), neuropeptide Y (NPY), and acetylcholine (Ach), which are released from autonomic nerve terminal varicosities, and become diffusely distributed to smooth muscle cells and endothelial cells in the vessel [73]. The effects of autonomic function and these neuropeptides on vascular functions including arterial tone may be mediated by nitric oxide (NO) and endothelin, vasoactive factors produced by endothelial cells [74]. Additionally, platelet aggregation might serve to mediate the effects of autonomic nervous dysfunction on progression of atherosclerosis [75].

Furthermore, autonomic nervous dysfunction disturbs metabolic factors and may be attributable to classical atherosclerotic risk factors, such as hypertension, dyslipidemia, and diabetes mellitus. Yamada and Katagiri showed that the autonomic nervous system plays an important role in communicating organ-to-organ metabolic information [76]. Using these systems, the brain obtains information regarding peripheral metabolic status and processes the signals to regulate peripheral metabolism. Indeed, a relationship between autonomic nervous function and metabolic syndrome was shown in a large prospective cohort study conducted over a 2-year period of follow-up examinations [77]. Their findings indicated that an index of autonomic dysfunction was associated with increased numbers of metabolic syndrome components, such as high blood pressure and low HDL cholesterol.

The effects of autonomic dysfunction on atherosclerosis may be mediated by endocrinological alterations, including circulating epinephrine [78], as well as insulin resistance and adipocytokines, including leptin. Recent findings in animal models clearly showed that leptin, which reduces food intake, is significantly associated with autonomic nervous function [79]. The leptin receptor is expressed in the central nervous system. In our study conducted with the HSCAA cohort and performed under clinical conditions, we showed an association between plasma leptin and autonomic nervous function in patients with type 2 diabetes, and that association was independent of other clinical factors [80]. Additionally, brain-derived neurotrophic factor (BDNF) may be another key factor related to the effects of autonomic nervous function [81], as it has critical roles in survival, growth, maintenance, and death of central and peripheral neurons, and is also involved in regulation of the autonomic nervous system. In the HSCAA cohort study, we found a positive and significant association between plasma BDNF and autonomic nervous function in patients with cardiovascular risk factors [16].

## 6. Fatigue, Atherosclerosis and Cardiovascular Diseases

Fatigue is a common symptom in patients with a variety of conditions. Although the pathophysiological details have not been well characterized, it is known to be associated with continuous short sleep deprivation and low sleep quality. Recent epidemiological studies have found that fatigue is more common in patients with cardiovascular risk factors, such as diabetes [82], obesity [83], and sleep apnea [84]. We showed that higher fatigue score, determined using a recently established fatigue questionnaire, is a predictor of cardiovascular events in patients undergoing hemodialysis, with an impact independent of the presence of diabetes or past history of cardiovascular diseases [85]. Although the underlying mechanisms of those findings are not clear at present, potential candidates include short sleep duration, low sleep quality, autonomic nervous dysfunction such as elevated sympathetic nervous function, and altered endocrine and immune functions. We reported that loading of psychological fatigue in rats was associated with disturbance of neuroendocrinological functions [86]. Furthermore, in the HSCAA cohort study, fatigue score was found to be closely associated with plasma leptin level in patients with cardiovascular risk factors [87]. The leptin receptor is expressed in the central nervous system and may be involved in cardiac autonomic nerve function [80]. We have also shown that low plasma BDNF level, another potential biomarker indicating fatigue, is independently associated with a reverse-dipper pattern of nocturnal blood pressure [16] and development of chronic kidney disease (CKD) [88], in which autonomic nervous dysfunction may be involved, at least in part. Additional studies are needed to explore the mechanisms related to the contributions of fatigue to progression of atherosclerosis and cardiovascular diseases via autonomic dysfunction.

## 7. Conclusions and Perspectives

**Figure 4:**
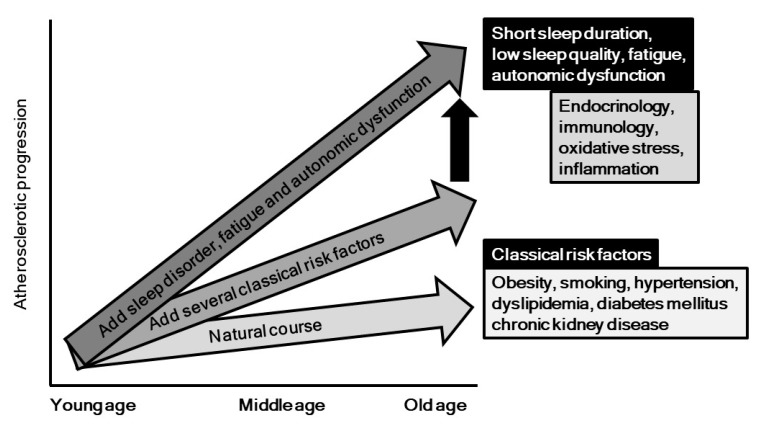
Potential impacts of psycho-behavior factors on progression of atherosclerosis. As shown in vertical black arrow, psycho-behavior factors might accelerate the degree of atherosclerotic progression, which is induced by classical risk factors such as diabetes, hypertension, obesity, and others.

Behavioral and psychosocial factors, especially sleep, have been gaining increased attention in regard to their relationship with development of cardiovascular disease. In this review, we aimed to focus on the pathogenic impact of sleep problems, autonomic nervous function, and fatigue in relation to development of atherosclerosis and cardiovascular diseases (Figure 4). It is possible that inflammatory and oxidative response, endothelial dysfunction, endocrinological and immunological factors, and metabolic responses, as well as yet unveiled mechanisms underlie the effects of sleep problems, autonomic dysfunction, and fatigue on atherosclerosis progression. As shown in Figure 4, these psycho-behavior factors might accelerate the degree of progression, which is induced by classical risk factors such as diabetes, hypertension, obesity, and others. As highlighted in this report, the association between sleep problems and atherosclerosis has mainly been demonstrated in healthy populations, and results of a prospective large cohort study of patients with atherosclerotic risks are definitely needed. Moreover, no randomized controlled trial to examine the impact of improvement of these psycho-behavior problems on progression of atherosclerosis and occurrence of cardiovascular diseases has been reported. Additionally, basic studies are mandatory to identify feasible biomarkers for assessment of sleep problems and autonomic dysfunction in clinical settings. Fortunately, greater attention and additional investigations have brought focus to this important research field, and we believe that behavior factors will be recognized in the near future as promising clinical targets for prevention of atherosclerotic diseases.

## Figures and Tables

**Table 1:** Association of subjective or objective sleep duration and quality with carotid IMT and baPWV in patients with atherosclerotic risk factors.

Surrogate Marker of Atherosclerosis	Sleep Parameter	Study Design	Subjects	Sleep Measurement	Comments	References
Carotid intima media thickness (IMT)	Duration	Cross-sectional	Elderly (*n* = 86)	PSQI Actigraphy	Shorter sleep duration (<5 h) increased IMT as compared to longer duration (>7 h)	Nakazaki et al. [19]
	Quality	Cross-sectional	Elderly (*n* = 86) Elderly (*n* = 201) Type 2 diabetes mellitus (*n* = 63) Cardiovascular risk factors (*n* = 330)	Self-reported questionnaire Single-channel EEG Actigraphy	Insomnia associated with IMT as compared with non-insomnia	Nakazaki et al. [19] Nagai et al. [20] Yoda et al. [21] Kadoya et al. [11]
	Apnea-hypopnea	Cross-sectional	Cardiovascular risk factors (*n* = 330)	Apnomonitor	OSA associated with IMT and plaque score	Kadoya et al. [11]
Brachial-ankle pulse wave velocity (baPWV)	Quality	Cross-sectional	Type 2 diabetes mellitus (*n* = 724)	PSQI	Poor sleep quality associated with higher PWV	Osonoi et al. [22]
		Prospective	Cardiovascular risk factors (*n* = 306)	Actigraphy	Low sleep quality associated with progression of PWV over 3-year period	Kadoya et al. [18]
	Apnea-hypopnea	Cross-sectional	Ischemic stroke (*n* = 127)	Polysomnography	OSA associated with PWV	Chen et al. [10]

PSQI: Pittsburg sleep quality index, EEG: electro-encephalography: OSA: obstructive sleep apnea.

**Table 2:** Associations of subjective or objective sleep duration, and quality with carotid IMT and baPWV in healthy populations.

Surrogate Marker of Atherosclerosis	Sleep Parameter	Study Design	Population Characteristics	Sleep Measurement	Comments	References
Carotid intima media thickness (IMT)	Duration	Cross-sectional	Healthy, middle-aged (*n* = 617)	Actigraphy	Shorter sleep duration (<5 h) increase IMT.	Sands et al. [23]
			General population (*n* = 2383) Health check-up subjects (*n* = 2214)		Long sleep duration (>7 h or >11 h) significantly correlated with the incidence of carotid artery atherosclerosis	Wolff et al. [24] Abe et al. [25]
Brachial-ankle pulse wave velocity (baPWV)	Duration	Cross-sectional	Health check-up subjects (*n* = 18,106)	PSQI	Subjective short sleep duration (<5 h) is associated with higher PWV	Kim et al. [28]
			Health check-up subjects (*n* = 18,106) Health check-up subjects (*n* = 4268) Health check-up subjects (*n* = 3508)	Self-Report questionnaire PSQI	Long sleep duration (> 8 h) is associated with elevated PWV	Kim et al. [28] Yoshioka et al. [29] Tsai et al. [30]
	Quality	Cross-sectional	Health check-up subjects (18,106)	PSQI	Poor sleep quality is associated with higher PWV	Kim et al. [28]

PSQI: Pittsburg sleep quality index.

**Table 3:** Multiple linear regression analyses of macro TSH and sleep parameters.

Variables	Sleep Physical Activity		% Sleep	
	β	*p*	β	*p*
Macro TSH (high = 1. Low = 0)	0.145	0.01	−0.150	<0.01
Adjusted *R*^2^	0.041	<0.01	0.047	<0.01

Multiple linear regression analyses were performed. Covariates in each model included age, male gender, body mass index, presence of hypertension, dyslipidemia, and diabetes mellitus. Higher sleep physical activity and lower % sleep each represent low sleep quality. TSH: thyroid-stimulating hormone. β: standard regression coefficient. Modified from [34].

**Table 4:** Associations of HRV with carotid IMT and baPWV.

Surrogate Marker of Atherosclerosis	Study Design	Population	Comments	References
Carotid intima media thickness (IMT)	Cross-sectional	Cardiovascular risk factors (*n* = 330) Depressed with cardiovascular risk factors (*n* = 391) Males >40 years old (*n* = 124) Cardiovascular risk factors (*n* = 101)	HRV associated with carotid IMT, independent of sleep quality and apnea-hypopnea. Inflammation may be involved in association between autonomic dysfunction and atherosclerosis.	Kadoya et al. [11] Pizzi et al. [69] Ulleryd et al. [70] Pereira et al. [71]
	Retrospective	Hypertensive (*n* = 200)	HRV associated with renal damage	Melillo et al. [68]
	Prospective	Type 2 diabetes (*n* = 61)	Decreased HRV may predict progression of carotid atherosclerosis	Gottsater et al. [67]
Brachial-ankle pulse wave velocity (baPWV)	Cross-sectional	Type 1 diabetes (*n* = 344)	Lower HRV associated with higher baPWV (no prospective studies available)	Jaiswal et al. [72]

HRV: heart rate variability.
